# SGLT2i relieve proteinuria in diabetic nephropathy patients potentially by inhibiting renal oxidative stress rather than through AGEs pathway

**DOI:** 10.1186/s13098-024-01280-5

**Published:** 2024-02-16

**Authors:** Xiao-chun Zeng, Yuan Tian, Xian-ming Liang, Xiao-bin Wu, Chun-meng Yao, Xiao-min Chen

**Affiliations:** 1 Department of Endocrinology and Metabolism, School of Medicine, Zhongshan Hospital of Xiamen University, Xiamen University, 201-209 Hubin South Road, 361004 Xiamen, P.R. China; 2 The School of Clinical Medicine, Fujian Medical University, 350004 Fuzhou, P.R. China; 3 Center of Clinical Laboratory, School of Medicine, Zhongshan Hospital of Xiamen University, Xiamen University, 201-209 Hubin South Road, 361004 Xiamen, P. R. China; 4 Department of Nephrology, School of Medicine, Zhongshan Hospital of Xiamen University, Xiamen University, 201-209 Hubin South Road, 361004 Xiamen, P. R. China

**Keywords:** SGLT2i, Diabetic nephropathy, Proteinuria, Oxidative stress, 8-OHdG, AGEs

## Abstract

**Aims:**

To estimate the effects of the sodium-glucose cotransporter 2 inhibitor (SGLT2i) on proteinuria and oxidative stress expression in type 2 diabetes patients.

**Materials and methods:**

68 patients with type 2 diabetes mellitus (T2DM) were divided into three groups according urinary albumin-to-creatinine ratio (UACR), including T2DM with non-albuminuria group (UACR < 30 mg/g), T2DM with microalbuminuria group (30 ≤ UACR ≤ 300 mg/g), T2DM with macroalbuminuria group (UACR>300 mg/g). They all received SGLT2 inhibitors (SGLT2i) treatment for 12 weeks. The expression of advanced glycation end products (AGEs) in plasma and 8-hydroxy-2-deoxyguanosine (8-OHdG) in urine were measured as indications of oxidative stress. The 24-hour urine samples were collected to measure the concentration of proteinuria and 8-OHdG before and after 12 weeks SGLT2i treatment. Plasma renin activity (PRA), angiotensin II (Ang II) and Aldosterone (ALD) were measured to evaluate renin angiotensin aldosterone system (RASS) levels.

**Results:**

After 12 weeks SGLT2 inhibitors treatment, the median values of 24-hour proteinuria decreased in macroalbuminuria compared to baseline (970 vs. 821 mg/d, *P* = 0.006). The median values of AGEs and 8-OHdG decreased in microalbuminuria and macroalbuminuria groups when compared to baseline, AGEs (777 vs. 136 ug/ml, *P* = 0.003) and (755 vs. 210 ug/ml, *P* = 0.001), 8-OHdG (8.00 vs. 1.88 ng/ml, *P* = 0.001) and (11.18 vs. 1.90 ng/ml, *P* < 0.001), respectively. Partial correlations showed that 8-OHdG were relevant to the baseline 24-h proteinuria (*r* = 0.389, *p* = 0.001), the reduction of OHdG (Δ8-OHdG) were positively correlated with the decrease of 24-h proteinuria (Δ24-h proteinuria) after 12 weeks of SGLT2i treatment (*r* = 0.283, *P* = 0.031). There was no significant correlation between 24-h proteinuria and AGEs in baseline (*r* = −0.059, *p* = 0.640) as well as between ΔAGEs and Δ24-h proteinuria (*r* = 0.022, *p* = 0.872) after12 weeks of SGLT2i treatment in T2DM patients.

**Conclusions:**

SGLT2i may reduce proteinuria in diabetic nephropathy patients, potentially by inhibiting renal oxidative stress, but not through the AGEs pathway and does not induce RAAS activation.

**Trial registration:**

This clinical trial was registered on 15/10/2019, in ClinicalTrials.gov, and the registry number is NCT04127084.

**Supplementary Information:**

The online version contains supplementary material available at 10.1186/s13098-024-01280-5.

## Introduction

As one of the most frequent and severe complications of diabetes mellitus (DM), diabetic nephropathy (DN) is an important cause of end-stage renal disease and is recognized as a public health problem worldwide. On a global scale, the incidence and prevalence of DN has dramatically risen over the past decades, especially in developing countries. The overall pooled prevalence of DN in China was 21.8% [1]. Complex and diverse factors are related to the development and progression of DN, including oxidative stress, hemodynamic abnormalities, metabolic disorders, and hormone synthesis disorders such as excess Ang II. Oxidative stress plays an important role in this process [2]. Reactive oxygen species (ROS) are necessary for normal cell signaling, but excess production of ROS can disrupt the cellular redox balance and overwhelm their antioxidant capacity, leading to oxidative stress [3]. The expression of NAD(P)H (Nicotinamide Adenine Dinucleotide Phosphate) oxidase subunits NOX4 and p22phox are upregulated in the kidney of diabetic rats, which suggest that continuous hyperglycemia can induce protein kinase C(PKC)-dependent activation of NAD(P)H oxidase, vascular NAD(P)H oxidase drives the production of ROS, these conditions will eventually contribute to diabetic nephropathy [4]. Diabetes causes inhibition of glucose-6-phosphate dehydrogenase via activation of PKA, and decreases NADPH, adequate supply of NADPH is the principal intracellular reductant for all cells, which contributes to oxidative stress in rat kidney cortex [5]. However, the precise mechanism of oxidative stress accelerating the development of diabetic nephropathy is still not completely clear.

ROS can cause oxidative modification of cellular macromolecules such as carbohydrates, lipids, proteins, and oxidative deoxyribonucleic acid (DNA) [6]. Advanced glycation end products (AGEs) are a group of complex oxidant compounds, which are slowly synthesized by non-enzymatic glycation between reducing sugars (such as glucose or fructose) and proteins or lipids, AGEs play an important role in the pathogenesis of diabetic vascular disease [7]. AGEs promote the development of DKD through the activation of oxidative stress, renin angiotensin aldosterone system (RAAS) and other multifactorial mechanisms and ultimately lead to characteristic lesions of DKD including glomerular basement membrane (GBM) thickening, extracellular matrix (ECM) accumulation, mesangial expansion, podocytes apoptosis, glomerulosclerosis, glomerular filtration reduction, interstitial fibrosis [8]. RAGE (receptor for advanced glycation end products) is the signal-transducing receptor for AGEs, Nakamura K et al. demonstrated that serum endogenous sRAGE (soluble form of RAGE) levels were significantly higher in type 2 diabetic patients than that of non-diabetes subjects [9]. More and more evidences show that AGEs are related to the pathophysiology of DKD. Koska J revealed that the increase of plasma AGEs was closely associated with the deterioration of albuminuria and creatinine clearance rate induced by diabetic nephropathy, which was independent of glycemic control, higher AGE scores are expected to be a predictor of long-term risk of developing DKD [10].

Similarly, the oxidation of deoxyguanosine will cause the accumulation of 8-OHdG, which is considered as the most sensitive and useful marker of DNA damage. Current evidence suggests that 8-OHdG lesions in DNA can lead to somatic mutation during cell replication, which may contribute to smooth muscle proliferation. Compared with normal control, 8-OHdG in patients with diabetes and even in the pre-diabetes group increased significantly [11]. 8-OHdG is excreted in urine which is stable and easy to collect, therefore, in recent years, 8-OHdG has been used to measure oxidative stress [12].

Mitochondria are the main source of ROS, and angiotensin induced significant increase of mitochondrial ROS [13]. NADPH oxidases are considered “professional ROS producers” as their sole role is to produce ROS [14]. Angiotensin II has been proved to be a pro-oxidant mainly stimulating NADPH oxidase (NOX) through G-protein-coupled receptor type 1 (ANG II type 1 receptor, AT1R) [15]. Studies have shown that the use of AT1R inhibitors can reduce ROS production in renal mitochondria. The protective effect of AT1R inhibitor on the kidney is independent of the reduction of both systolic blood pressure and blood glucose levels [16], which may be related to the inhibition of renal oxidative stress.

Sodium-glucose cotransporter 2 inhibitors (SGLT2i) are relatively new type of hypoglycemic drugs, we have demonstrated that SGLT2i alleviate nephrin loss and enhance TGF-β1 excretion in urine in T2DM with albuminuria. Our findings suggest that the anti-albuminuria effect of SGLT2 inhibitors could be attributed to mitigating podocyte apoptosis, attenuating renal fibrosis and promoting weight loss [17]. The antioxidant capacity of SGLT2i has been a research hotspot in recent years. However, most studies were conducted in cultured cells and animal models [18–20], rarely involved changes of oxidative stress in patients with type 2 diabetic nephropathy [21]. We attempted to investigate the effect of SGLT2i on proteinuria and its relationship with oxidative stress and RAAS.

## Materials and methods

### Study design

The trial was a 12-week, randomized, blank-controlled clinical trial including persons with T2DM (*n* = 68) treated in the department of endocrinology or nephrology of Zhongshan Hospital of Xiamen University and the controls (*n* = 10) between October 2019 and October 2020. The inclusion criteria for the study groups were patients who met the World Health Organization criteria for type 2 diabetes, age between 18 and 80, with any antidiabetic drugs except SGLT2 inhibitors or GLP-1RA, without using angiotensin receptor blocker (ACEI/ARB) within 1 month. The key exclusion criteria for the study groups were type 1 diabetes, acute or chronic glomerulonephritis, long term use of corticosteroids or immunosuppressants, impaired liver function, impaired renal function (eGFR < 45 ml/min/1.73 m2), recent acute illness (including diabetic ketoacidosis and hyperglycaemic hyperosmolar coma, acute infection, acute heart failure, stroke), women in pregnancy or lactation. Sixty-eight participants were divided into three groups based on their urinary albumin/creatinine ratio (UACR): non-albuminuria group (UACR < 30 mg/g) (*n* = 21), microalbuminuria group (30 ≤ UACR ≤ 300 mg/g) (*n* = 20), and macroalbuminuria group (UACR>300 mg/g) (*n* = 27). 68 diabetic patients were randomized (1:1:1) by computer-generated numbers to treated with dapaglifozin, empaglifozin or canaglifozin for 12 weeks. Follow-up visits were made every two weeks. The study was approved by the Ethics Committee of Zhongshan Hospital of Xiamen University and was registered at ClinicalTrials.gov (NCT04127084). Informed consent was obtained from all participants [17].

### Primary and secondary endpoints

The primary endpoints were changes of 24-hour proteinuria, 8-OHdG, AGEs after 12-week SGLT2i treatment. The secondary endpoints included changes of ALD, Ang II and PRA after 12-week SGLT2i treatment.

### Laboratory procedures

Fasting blood and 24-hour urine were collected from healthy participants when they were enrolled. Similarly, fasting blood and 24-hour urine of enrolled diabetes patients were collected before and after 12 weeks SGLT2i treatment. Weight, waist circumference (WC) and hip circumference (HC) were recorded at the same time. The 24-hour urine samples were collected to measure the concentration of proteinuria and 8-OHdG. The blood was drawn to measure AGEs, HbA1c, Scr, Ang II, blood lipids, lipoprotein, and uric acid.

Blood lipid and lipoprotein concentrations, including triglyceride (TG), total cholesterol (TC), low-density lipoprotein cholesterol (LDL-C) and high-density lipoprotein cholesterol (HDL-C), were measured by standard methods (Roche CoBAS8000 automatic biochemical analyzer). Glomerular filtration rate (eGFR) was calculated according to CKD-EPI creatinine equation. Uric acid (UA) and serum creatinine (Scr) were determined by uricase-peroxidase method and picric acid method respectively by automatic method (Beckman Coulter Biochemical analyzer, USA). HbA1c was determined by high performance liquid chromatography (HPLC) (Bio-RAD, Inc., Hercules, CA, USA). Urinary albumin was determined by immunoturbidimetry and creatinine was determined by picric acid method (Roche C701 automatic biochemical analyzer). 8-OHdG (Japan Institute for the Control of Aging, JaICA) and AGEs (CELL BIOLABS, INC) was detected by enzyme-linked immunosorbent assay (ELISA). Plasma renin activity (PRA) was assayed by direct concentration method, angiotensin II (Ang II) and Aldosterone (ALD) were determined by chemiluminescent immunoassay (Autobio Diagnostics Co, Ltd.). The data of Weight, WC, HC, HbA1c, Scr, TG, TC, LDL-C, HDL, UA had been reported [17].

### Statistical analysis

The normal distribution data are presented as the mean ± standard deviation. The skewed distribution variables are expressed as the median (interquartile rang, IQR). Basic characteristics were compared by one-way ANOVA or rank-sum test according to whether the data were normally distributed. The basic characteristics were compared with the data after treatment by paired t test or paired rank sum test according to whether the data were normally distributed. Partial correlation was calculated to explore the relationships between proteinuria and 8-OHdG, AGEs with imposing control in confounding factors. Data analysis was performed using IBM SPSS Statistics 25.0 and GraphPad Prism 8.0. *P* values <0.05 was considered significant.

## Results

### Comparisons of baseline characteristics of the study participants

In the baseline analysis, diabetic duration, the proportion of gender, TC, TG, HDL-C, UA, PRA, Ang II, ALD and AGEs among the four groups have no statistically difference. There were significant differences in BW, WC, HbA1c, DBP between the diabetes patients and the control group. Patients with albuminuria were elder than those without albuminuria, who also had longer duration and higher SBP. Patients with macroalbuminuria had significant decreases in eGFR compared with the control group and the patients without albuminuria. The median values of 8-OHdG at baseline (4.50, 8.00, 11.18 ng/ml) in the diabetic patients gradually increased with the increase of UACR, although the controls showed the highest urinary 8-OHdG at baseline. The median values of baseline AGEs (775, 777, 755 ug/ml) are similiar in the three diabetic groups, as shown in Table 1. The percentage of metformin and dipeptidyl peptidase-IV inhibitors (DPP-IV inhibitors) in the baseline analysis were not statistically significant between different UACR levels, as shown in Table 1. Three types of SGLT2 inhibitors, including dapagliflozin, empagliflozin, and canagliflozin, of which 22 (32.4%) used dapagliflozin, 27 (39.7%) used empagliflozin, and 19 (27.9%) used canagliflozin, were randomized to administer throughout the study. The percentage of SGLT2 inhibitor initiation was not statistically significant (*p* = 0.897) [17].

**Table 1:** Comparisons of baseline characteristics in all participants

Variable	Control group	UACR < 30 mg/g	30 ≤ UACR ≤ 300 mg/g	UACR>300 mg/g	P-value
	*n* = 10	*n* = 21	*n* = 20	*n* = 27	
Male/female (n)	6/4	13/8	12/8	16/11	0.998
Age (year)	45 ± 17	45 ± 13	59 ± 13^ab^	58 ± 12^ab^	0.001
duration (year)	NA	4 (2, 8)	8 (4, 11)	8 (4, 11)	0.071
**Medicine (%)**					
Metformin	NA	12/21 (57%)	9/20 (45%)	14/27 (52%)	0.738
DPP-IV inhibitor	NA	6/21 (29%)	5/20 (25%)	7/27 (26%)	0.964
BW (kg)	61.15 ± 8.01	76.50 ± 12.67^a^	72.33±16.07^a^	68.63±14.58	0.012
WC (cm)	76±8	88±9^a^	91±11^a^	87±11^a^	0.003
HC (cm)	90±5	97±6^a^	98±10^a^	94±8	0.003
HbA1c (%)	5.38±0.41	7.53±1.41^a^	8.31±1.38^a^	7.90±1.78^a^	<0.001
TC (mmol/L)	4.85±0.68	5.06 ±1.03	4.36±0.78	5.20±1.32	0.100
TG (mmol/L)	1.21 (0.65, 1.55)	1.54 (1.08, 2.31)	1.69 (1.20, 2.03)	1.34 (1.17, 2.45)	0.311
LDL-C (mmol/L)	3.05±0.66	3.21±0.73	2.67±0.59^b^	3.34±0.86^c^	0.036
HDL-C (mmol/L)	1.38±0.40	1.13±0.16	1.21±0.29	1.15±0.23	0.311
Scr (umol/L)	67±15	78±14	74±16	99±42^ac^	0.035
UA (umol/L)	340±92	358±100	347±101	395±87	0.329
eGFR (ml/min/1.73 m^2^)	109±22	99±21	88±20	74±24^ab^	<0.001
24-h proterinuria (mg/d)	76 (53, 101)	88 (77, 111)	183 (132, 226)^ab^	970 (676, 2345)^abc^	0.000
UACR (mg/g)	3 (1, 4)	6 (4, 12)	87 (42, 203)^ab^	861 (459, 1966)^abc^	<0.001
SBP (mmHg)	116±13	128±14	135±17^a^	138 ±17^a^	0.002
DBP (mmHg)	66±8	79±12^a^	79±11^a^	78±9^a^	0.010
ALD (pg/ml)	173 (128, 260)	168 (120, 220)	176 (128, 225)	163 (121, 223)	0.954
Ang II (pg/ml)	98 (81, 119)	98 (83, 111)	90 (86, 124)	109 (88, 125)	0.550
PRA (ng/ml/h)	2.75 (1.00, 5.82)	1.38 (0.51, 2.86)	0.86 (0.33, 2.59)	0.68 (0.21, 2.50)	0.122
8-OHdG (ng/mL)	12.15 (6.03, 17.56)	4.50 (2.31, 10.39)^a^	8.00 (2.55, 9.38)	11.18 (6.40, 13.42)^b^	0.005
AGEs (ug/mL)	433 (153, 882)	775 (376, 932)	777 (147, 1160)	755 (413, 933)	0.599

Data are expressed as mean ± standard deviation or median (interquartile rang)

^a^Versus Control Group, *P*<0.05

^b^Versus UACR<30 mg/g Group, *P*<0.05

^c^Versus 30≤UACR≤300 mg/g Group, *P*<0.05

*Abbreviations* UACR: urinary albumin/creatinine ratio; BW: body weight; WC: Waist Circumference; HC: Hip Circumference; HbA1c: Glycated hemoglobin; TC: Total choles-terol; TG: triglycerides; LDL-C: low-density lipoprotein cholesterol; HDL-C: high-density lipoprotein cholesterol; Scr: serum creatinine; UA: Uric acid; eGFR: estimated Glomerular Filtration Rate; SBP: Systolic blood pressure; DBP: Diatolic blood pressure; ALD: Aldosterone; Ang II: angiotensin II; PRA: plasma renin activity; 8-OHdG: 8-hydroxy-2- deoxyguanosine; AGEs: advanced glycation end products

### Effect of SGLT2i on 24-hour proteinuria and HbA1c

**Fig. 1:**
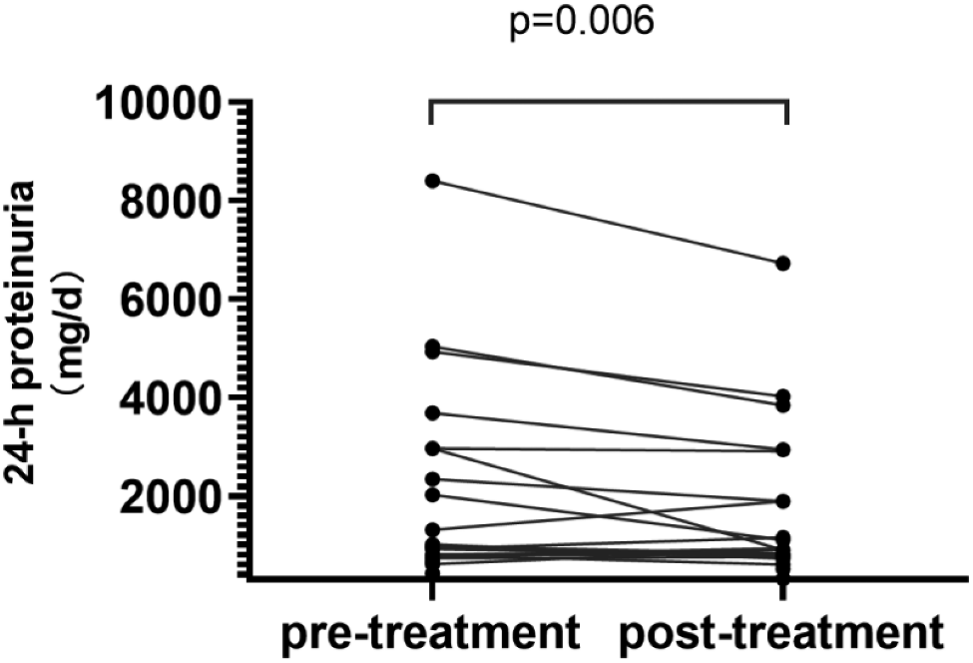
Changes in 24-h proteinuria from baseline to week 12 in T2DM with macroalbuminuria group (UACR>300 mg/g)

After 12-week SGLT2i treatment, the 24-hour proteinuria levels decreased in macroalbuminuria groups (970 [676, 2345] vs. 821 [273, 1893] mg/d, *P* = 0.006), as shown in Fig. 1. The median value of HbA1c decreased in all groups, but only the reduction of HbA1c in the microalbuminuria group was statistically significant ( [8.31 ± 1.38] vs. [7.46 ± 1.16] %, *p* = 0.042).

### 8-OHdG and AGEs levels dramatically decreased in response to SGLT2 inhibitor treatment

**Fig. 2:**
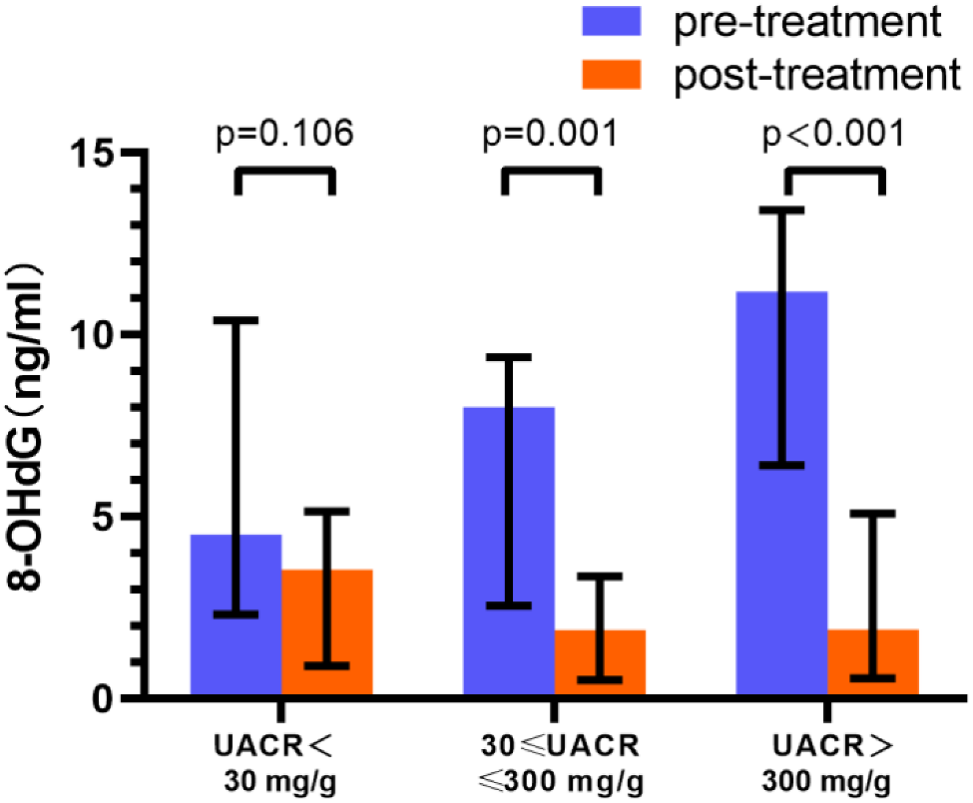
Changes of 8-OHdG before and after 12-week SGLT2 inhibitors treatment in different UACR levels. 8-OHdG: 8-hydroxy-2-deoxyguanosine

**Fig. 3:**
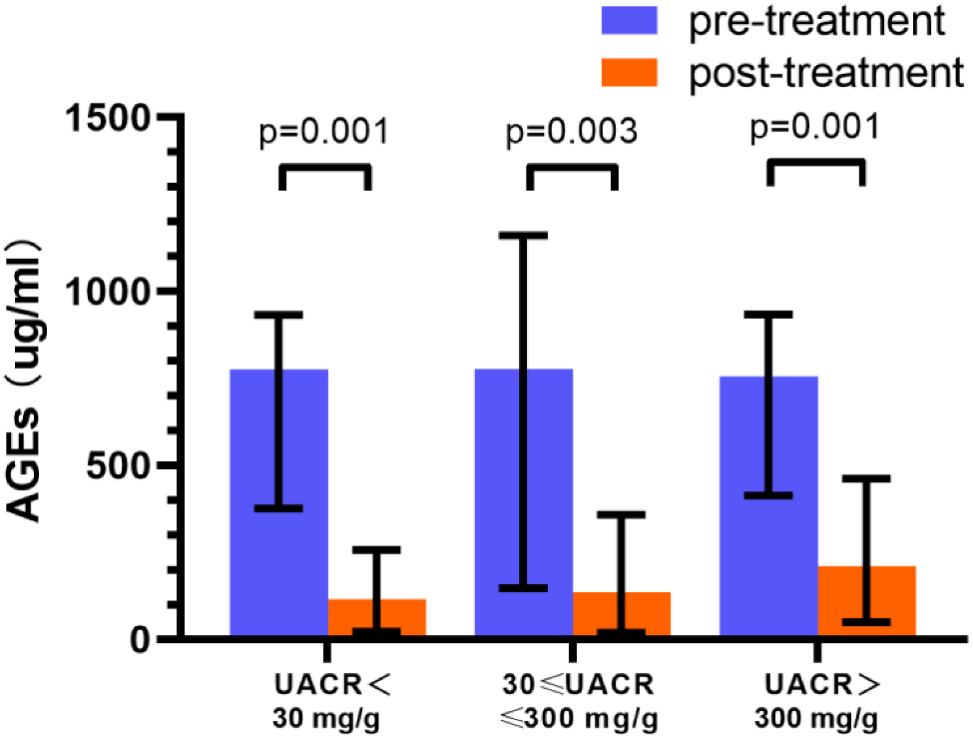
Changes of AGEs before and after 12-week SGLT2 inhibitors treatment in different UACR levels. AGEs: advanced glycation end products

After 12 weeks of SGLT2 inhibitor treatment, 8-OHdG significantly decreased in the microalbuminuria and macroalbuminuria groups compared with baseline (8.00 [2.55, 9.38] vs. 1.88 [0.50, 3.36] ng/ml, *P* = 0.001) and (11.18 [6.40, 13.42] vs. 1.90 [0.55, 5.08] ng/ml, *P* < 0.001), respectively, as shown in Fig. 2 and Table 2. After 12 weeks of SGLT2 inhibitor treatment, AGEs dramatically decreased in three groups compared to baseline, which were (775 [376, 932] vs. 115 [22, 257] ug/ml, *P* = 0.001, 777 [147, 1160] vs. 136 [19, 358] ug/ml, *P* = 0.003), and (755 [413, 933] vs. 210 [49, 462] ug/ml, *P* = 0.001), respectively, as shown in Fig. 3 and Table 2.

**Table 2:** Comparisons of parameters before and after 12-week treatment in type 2 diabetes

Variable	UACR<30 mg/g			30≤UACR ≤300 mg/g			UACR>300 mg/g		
	Pre-treatment	Post-treatment	p-value	Pre-treatment	Post-treatment	p-value	Pre-treatment	Post-treatment	p-value
BW (kg)	76.50±12.67	73.04±12.55	<0.001	72.33±16.07	69.50±15.39	<0.001	68.63±14.58	65.84±14.12	<0.001
WC (cm)	88±9	84±8	<0.001	91±11	87±10	<0.001	87±11	84±11	<0.001
HC (cm)	97±6	95±7	0.068	98±10	95±8	0.001	94±8	91±7	<0.001
HbA1c (%)	7.53±1.41	7.12±1.12	0.054	8.31±1.38	7.46±1.16	0.042	7.90±1.78	7.43±1.27	0.150
TC (mmol/L)	5.06 ±1.03	5.03±0.94	0.894	4.36±0.78	4.49±1.09	0.480	5.20±1.32	4.91±1.13	0.269
TG (mmol/L)	1.54 (1.08, 2.31)	1.35 (1.01, 2.05)	0.191	1.69 (1.20, 2.03)	1.41 (0.92, 1.93)	0.028	1.34 (1.17, 2.45)	1.62 (1.13, 2.64)	0.638
LDL-C (mmol/L)	3.21±0.73	3.33±0.74	0.476	2.67±0.59	2.85±0.82	0.118	3.34±0.86	3.21±0.84	0.433
HDL-C (mmol/L)	1.13±0.16	1.18±0.13	0.021	1.21±0.29	1.24±0.24	0.320	1.15±0.23	1.17±0.24	0.493
Scr (umol/L)	78±14	74±14	0.137	74±16	73±18	0.920	99±42	100±39	0.563
UA (umol/L)	358±100	327±86	0.048	347±101	367±103	0.481	395±87	372±70	0.156
eGFR (ml/min/1.73m^2^)	99±21	103±19	0.156	88±20	87±22	0.764	74±24	70±21	0.090
24-h proteinuria (mg/d)	88 (77, 111)	119 (81, 171)	0.079	183 (132, 226)	193 (127, 269)	0.983	970 (676, 2345)	821 (273, 1893)	0.006
UACR (mg/g)	6 (4, 12)	6 (4, 10)	0.848	87 (42, 203)	75 (21, 152)	0.003	861 (459, 1966)	738 (213, 1482)	0.037
SBP (mmHg)	128±14	123±14	0.148	135±17	130±15	0.146	138 ±17	134±15	0.126
DBP (mmHg)	79±12	77±12	0.258	79±11	73±10	0.018	78±9	76±11	0.506
ALD (pg/ml)	168 (120, 220)	182 (142, 255)	0.526	176 (128, 225)	197 (153, 277)	0.263	163 (121, 223)	168 (122, 240)	0.755
Ang II (pg/ml)	98 (83, 111)	96 (81, 138)	0.259	90 (86, 124)	97 (81, 174)	0.737	109 (88, 125)	107 (90, 194)	0.639
PRA (ng/ml/h)	1.38 (0.51, 2.86)	3.15 (1.20, 4.91)	0.211	0.86 (0.33, 2.59)	0.84 (0.25, 2.37)	0.926	0.68 (0.21, 2.50)	0.63 (0.24, 2.86)	0.614
8-OHdG (ng/mL)	4.50 (2.31, 10.39)	3.35 (0.89, 5.14)	0.106	8.00 (2.55, 9.38)	1.88 (0.50, 3.36)	0.001	11.18 (6.40, 13.42)	1.90 (0.55, 5.08)	<0.001
AGEs (ug/mL)	775 (376, 932)	115 (22, 257)	0.001	777 (147, 1160)	136 (19, 358)	0.003	755 (413, 933)	210 (49, 462)	0.001

Data are expressed as mean±standard deviation or median (interquartile rang)

*Abbreviations* UACR: urinary albumin/creatinine ratio; BW: body weight; WC: Waist Circumference; HC: Hip Circumference; HbA1c: Glycated hemoglobin; TC: Total choles-terol; TG: triglycerides; LDL-C: low-density lipoprotein cholesterol; HDL-C: high-density lipoprotein cholesterol; Scr: serum creatinine; UA: Uric acid; eGFR: estimated Glomerular Filtration Rate; 24-h proteinuria: 24-hour Proteinuria; SBP:Systolic blood pressure; DBP: Diatolic blood pressure; ALD: Aldosterone; Ang II: angiotensin II; PRA: plasma renin activity; 8-OHdG: 8-hydroxy-2- deoxyguanosine; AGEs: advanced glycation end products

### Correlations of 24-hour proteinuria with changes of 8-OHdG and AGEs

After imposing control in confounding factors (age, body weight, HC, WC, HbA1c, LDL-C, SBP, DBP, Scr, eGFR) in the partial correlation matrix, we found a significant correlation between 8-OHdG and baseline 24-h proteinuria (*r* = 0.389, *p* = 0.001), while AGEs was not correlated with baseline 24 h-proteinuria (*r* = −0.059, *p* = 0.640), as shown in Table S1. Furthermore, after controlling for confounding factors including ΔBW, ΔWC, ΔHC, ΔDBP, ΔUA, ΔHDL, ΔTG, ΔHbA1c, the reduction of 8-OHdG (Δ8-OHdG) was positively correlated with the decrease of 24 h-proteinuria (Δ24 h-proteinuria) (*r* = 0.283, *P* = 0.031), and there was no significant correlation between changes in AGEs (ΔAGEs) and Δ24h-proteinuria (*r* = 0.022, *p* = 0.872), as shown in Table S2.

### SGLT2 inhibitors treatment did not induce the RAAS activation

There was no difference in medians values of PRA (2.75, 1.38, 0.86, 0.68 ng/ml/h, *P* = 0.122), Ang II (98, 98, 90, 109 pg/ml, *P* = 0.550), and ALD (173, 168, 176, 163 pg/ml, *P* = 0.954) at baseline among four groups, as shown in Table 1. There was no significant difference in renin angiotensin aldosterone system before and after SGLT2i treatment in non-albuminuria group, microalbuminuria and macroalbuminuria groups, PRA (1.38 [0.51, 2.86] vs. 3.15 [1.20, 4.91] ng/ml/h, *P* = 0.211), (0.86 [0.33, 2.59] vs. 0.84 [0.25, 2.37] ng/ml/h, *P* = 0.926), and (0.68 [0.21, 2.50] vs. 0.63 [0.24, 2.86] ng/ml/h, *P* = 0.614), respectively. Ang II (98 [83, 111] vs. 96 [81, 138] pg/ml, *P* = 0.259), (90 [86, 124] vs. 97 [81, 174] pg/ml, *P* = 0.737), and (109 [88, 125] vs. 107 [90, 194] pg/ml, *P* = 0.639), respectively. ALD (168 [120, 220] vs.182 [142, 255] pg/ml, *P* = 0.526), (176 [128, 225] vs. 197 [153, 277] pg/ml, *P* = 0.263), and (163 [121, 223] vs. 168 [122, 240] pg/ml, *P* = 0.755), respectively. After SGLT2i treatment, PRA in non-albuminuria group increased about two folds (1.38 [0.51, 2.86] vs. 3.15 [1.20, 4.91] ng/ml/h, *P* = 0.211), but there was not statistical significance, as shown in Table 2.

## Discussion

The incidence of type 2 diabetes (T2D) has been increasing worldwide, and DN remains one of the leading long-term complications of T2D [1]. The occurrence of proteinuria is the characteristic of diabetic kidney disease (DKD), effectively reducing proteinuria is extremely important and can prevent the progress toward end-stage renal disease. However, apart from renin-angiotensin-aldosterone blockers, there were few breakthroughs about treatment for diabetic nephropathy in recent years, beta-blockers and statins have not been shown to significantly improve the pathophysiology of diabetic nephropathy. Large-scale clinical trials have not only demonstrated the cardiovascular safety of SGLT2i, but also unexpectedly found that it could delay the progression of diabetic nephropathy. The renal and cardiac protective effects of SGLT2i were independent of glucose lowering effects [22], but the exact mechanism remains unclear. Traditionally, diabetic nephropathy (DN) has been considered “metabolic” nephropathy, distinct from “inflammatory” nephropathy, such as primary and secondary glomerular kidney diseases (many of which are termed “nephritis” to emphasize their inflammation) [23]. Now, the diabetic environment is known to activate oxidative stress and inflammation in the kidneys. Oxidative stress plays a key role in the development of diabetic kidney disease. Metabolic abnormalities in diabetes lead to overproduction of mitochondrial superoxide, which lead to activation of several major pathways involved in the pathogenesis of diabetic complications: increased formation of AGEs, increased expression of receptors for AGEs and their activating ligands, activation of protein kinase C isoforms, activation of NOX [24, 25]. We speculate that oxidative stress plays a critical role in the pathogenesis of diabetic nephropathy, antioxidant capacity is involved in the mechanism of SGLT2i on proteinuria.

8-OHdG is considered as a biomarker of DNA oxidative damage and one of the most widely studied oxidative metabolites [26], which was first reported by Kasai and Nishimura in 1984 [27]. Several studies have confirmed the close relationship between 8-OHdG and proteinuria in type 2 diabetes patients [28, 29], for example, Nishikawa et al. reported that the urinary 8-OHdG level of type 2 diabetes patients with albuminuria increased 1.9 times compared with patients without nephropathy [30]. The present study, we not only observed the decrease of proteinuria and 8-OHdG after SGLT2i treatment, but also found that SGLT2i potentially relieve proteinuria through reducing renal oxidative stress. After imposing control in confounding factors (age, body weight, HC, WC, HbA1c, LDL-C, SBP, DBP, Scr, eGFR) in the partial correlation matrix, we found a significant correlation between 8-OHdG and baseline 24-h proteinuria. Furthermore, the reduction of 8-OHdG (Δ8-OHdG) was positively correlated with the decrease of 24 h-proteinuria (Δ24h- proteinuria) after controlling the effects of decrease of blood pressure, blood glucose, blood lipids, uric acid and weight loss. Our results confirmed that SGLT2i can effectively relieve proteinuria by down-regulating oxidative stress, which may be related to the inhibition of Na-H exchanger (NHE). NHE is a family of ion transport pump proteins that exist on the surface of cell membranes, its inhibitors have direct anti-ROS effects in myocardial mitochondria [31]. Na+/H + exchanger isoform 3 (NHE3) contributes to Na+/bicarbonate reabsorption and ammonium secretion in early proximal tubules. Higher renal phosphoenolpyruvate carboxykinase expression in knockdown of NHE3 in renal tubules in Akita diabetic mice was associated with lower Na+-glucose cotransporter (SGLT)2 and higher SGLT1 expression, indicating a downward tubular shift in Na + and glucose reabsorption. NHE3-KO was associated with lesser kidney weight and eGFR and prevented diabetes-associated albuminuria [32]. Uthman L further demonstrated that empagliflozin (EMPA) attenuated TNF-α-induced ROS generation through NHE inhibition and lowering of cytoplasmatic Na + in human endothelial cells, which suggested that SGLT2i could reduce oxidative stress through inhibiting the novel /NHE/[Na+]c/ROS inflammation pathway [33]. SGLT-2i ameliorate the barrier dysfunction of human coronary artery endothelial cells under enhanced stretch through inhibiting NHE1 and scavenging of ROS [34].

Advanced glycation end products (AGEs) are long-lived chemical intermediates formed by reactions of chemically reactive sugars with proteins, lipids, and nucleic acids [10]. AGEs are metabolic mediators of kidney damage, and its correspondent receptor RAGE are widely expressed in podocytes, tubular epithelial cells and mesangial cells of the kidney. In persistent hyperglycemic state, AGEs/RAGE interaction triggers several signaling cascades such as IKK/NF-κB, ERK/MAPK, PKC, JNK and JAK/STAT and activates transcription factors such as NF-κB, CREB, AP-1, and STAT3, leading to ROS generation and amplifying inflammatory responses, which will further exacerbate the diabetic complication [35]. In the past, AGEs inhibitors such as aminoguanidine, pyridoxamine and alagebrium were considered to have renal protective effects. However, due to side effects and other reasons, research has been stagnant [36–38]. In our study, the median values of baseline AGEs are similiar in the three diabetic groups, after 12 weeks of SGLT2 inhibitor treatment, AGEs dramatically decreased in three groups compared to baseline. but unexpectedly, the decline of AGEs is not significantly related to the decrease of 24 h- proteinuria. We speculate that SGLT2i may relieve albuminuria in diabetic nephropathy patients potentially by inhibiting renal oxidative stress but not through AGEs pathway.

Possible mechanisms for proteinuria in diabetic patients include: impaired reabsorption by the epithelial cells of the proximal tubular and damaged glomerular filtration barrier. Elevated ROS levels can lead to mitochondrial dysfunction, bioenergetics defects, altered gene expression and cell death. Proximal tubular cells (PTCs) have high mitochondrial density that makes them susceptible to ROS-induced cell damage [39]. Continuous and extensive microvilli brush border cover the apical portion of the PTCs, the protein megalin is localized in the brush border microvilli. Megalin is involved in the mechanism of albumin reabsorption directly as a receptor. Studies have shown that ameliorating oxidative stress in renal tubules can up-regulate expression of megalin and albumin uptake of renal tubule [40, 41]. In diabetic rat models studied by Raysa S. Farias, SGLT2i ameliorates megalin-mediated protein reabsorption and reduce albuminuria [42]. Podocyte apoptosis plays a central and critical role in the disruption of the structural and functional integrity of the glomerular filtration barrier [43]. Podocytes are especially vulnerable to oxidative stress, podocyte loss leads to proteinuria [44]. SGLT2i has been shown to reduce proteinuria by alleviating podocyte apoptosis [17, 45]. Therefore, we speculate that SGLT2i could repair damage of PTCs and alleviate podocytes shedding by inhibiting renal oxidative stress, and further relieve proteinuria.

The hypovolemia caused by the diuretic action of SGLT2 inhibitors may result in the activation of systemic RAAS, and the activation of RAAS is associated with the increase of renal oxidative stress. Therefore, whether the application of SGLT2i will cause RAAS activation is one of our concerns. RAAS has been proved to be a pro-oxidant and its inappropriate activation contributes to the development of cardiovascular and renal diseases [46]. Our data suggested that application of SGLT2i did not activate the systemic RAAS. Macula densa (MD) senses the concentration of sodium chloride (NaCl) in the tubule fluid and regulate the perfusion pressure of afferent arterioles. This mechanism is defined as tubular glomerular feedback [47]. In diabetic kidney, the increase of SGLT2 transportation leads to the decrease of NaCl concentration transporting through macula densa, which will further lead to vasodilation of afferent arteriolar. SGLT2 inhibitors increases the concentration of NaCl in the tubular fluid flowing through the macula densa, leading to constriction of the afferent arterioles, which can correct the hyperfiltration of early diabetic nephropathy [48]. In the isolated tubular segments from rabbit kidneys, the increase of perfusate NaCl concentration to macula densa was proved to be associated with the inhibition of renin secretion [49]. Therefore, we infer that this may be the reason why SGLT2i does not cause RAAS activation even if it causes hypovolemia.

The strengths of this study are that it is the first to demonstrate that SGLT2 inhibitors may reduce proteinuria in patients with diabetes nephropathy potentially by inhibiting renal oxidative stress rather than by AGEs pathway and will not induce RAAS activation. However, this study has certain limitations. First of all, its sample size is relatively small, the research period is relatively short, and there is no active control group. Secondly, it is necessary to further investigate the internal mechanisms of SGLT2i in attenuating oxidative stress, mitigating inflammatory and inhibiting glomerular fibrosis through renal pathological biopsy.

## Conclusions

SGLT2i may alleviate proteinuria in diabetic nephropathy patients potentially by inhibiting renal oxidative stress, rather than through the AGEs pathway and does not induce RAAS activation. Our findings bring new evidence that that SGLT2 inhibitors should be regarded as one of the most promising antioxidants in the treatment of diabetic nephropathy.

### Electronic supplementary material

Below is the link to the electronic supplementary material.

Supplementary Material 1

Supplementary Material 2

## Data Availability

The datasets used or analyzed during the current study are available from the corresponding author upon reasonable request.

## Abbreviations

SGLT2i: Sodium-glucose cotransporter 2 inhibitors
T2DM: type 2 diabetes mellitus
UACR: urinary albumin/creatinine ratio
AGEs: advanced glycation end products
8-OHdG: 8-hydroxy-2deoxyguanosine
PRA: plasma renin activity
Ang II: angiotensin II
ALD: Aldosterone
RAAS: renin angiotensin aldosterone system
HbA1c: Glycated hemoglobin
DKD: diabetic kidney disease
DN: diabetic nephropathy
ROS: reactive oxygen species
PKA: protein kinase A
NAD (P) H: Nicotinamide Adenine Dinucleotide Phosphate
NOX: NADPH oxidases
RAGE: receptor for advanced glycation end products
sRAGE: soluble form of RAGE
DNA: deoxyribonucleic acid
AT1R: Ang II type 1 receptor
DPP-IV inhibitors: dipeptidyl peptidase-IV inhibitors
WC: waist circumference
HP: hip circumference
TG: triglyceride
TC: total cholesterol
LDL-C: low-density lipoprotein cholesterol
HDL-C: high-density lipoprotein cholesterol
eGFR: Glomerular filtration rate
UA: Uric acid
Scr: serum creatinine
NHE: Na-H exchanger
IKK: IκB kinase
NF-κB: nuclear factor kappa B
ERK/MAPK: extracellular signal-regulated kinase mitogen-activated protein kinase
PKC: protein kinase C
JNK: Jun N-terminal kinase
JAK/STAT: janus kinase/signal transducer and activator of transcription
CREB: cAMP-response element binding protein
AP-1: activator protein 1
PTCs: proximal tubular cells
NaCl: sodium chloride
MD: macula densa
