# Supplementary material for: SGLT2i relieve proteinuria in diabetic nephropathy patients potentially by inhibiting renal oxidative stress rather than through AGEs pathway
Source: Diabetol Metab Syndr. 2024 Feb 16;16:46. doi: 10.1186/s13098-024-01280-5 (PMC10870536; doi:10.1186/s13098-024-01280-5)
Supplement: Supplementary file 1 — Supplementary Material 1 [file 13098_2024_1280_MOESM1_ESM.doc]

**Table S1.** **Partial correlation analysis in 24-hour proteinuria and 8-OHdG, AGEs in baseline＊**

| **Variables** | **Correlation coefficient with**  **24-h proteinuria (*r*)** | ***P*-value** |
| --- | --- | --- |
| 8-OHdG | 0.389 | 0.001 |
| AGEs | －0.059 | 0.640 |

**^＊^**control the confounding factors age, body weight, HC, WC, HbA1c, LDL-C, SBP, DBP, Scr, eGFR

8-OHdG: 8-hydroxy-2- deoxyguanosine; AGEs: advanced glycation end products; HC: Hip Circumference; WC: Waist Circumference; HbA1c: Glycated hemoglobin; LDL-C: low-density lipoprotein cholesterol; SBP: Systolic blood pressure; DBP: Diatolic blood pressure; Scr: serum creatinine; eGFR: estimated Glomerular Filtration Rate;
