# Supplementary material for: SGLT2i relieve proteinuria in diabetic nephropathy patients potentially by inhibiting renal oxidative stress rather than through AGEs pathway
Source: Diabetol Metab Syndr. 2024 Feb 16;16:46. doi: 10.1186/s13098-024-01280-5 (PMC10870536; doi:10.1186/s13098-024-01280-5)
Supplement: Supplementary file 2 — Supplementary Material 2 [file 13098_2024_1280_MOESM2_ESM.doc]

**Table S2.** Partial correlation analysis between the decrease of 24-hour proteinuria with the changes of 8-OHdG and AGEs**^＊^**

| **Variables** | **Correlation coefficient**  **with** Δ24-h proteinuria **(*r*)** | ***P*-value** |
| --- | --- | --- |
| Δ 8-OHdG | 0.283 | 0.031 |
| Δ AGEs | 0.022 | 0.872 |

Deltas(Δ) are presented as the difference of variables before and after treatment

**^＊^**control the confounding factors ΔBW, ΔWC, ΔHC, ΔDBP, ΔUA, ΔHDL, ΔTG, ΔHbA1c

8-OHdG: 8-hydroxy-2- deoxyguanosine; AGEs: advanced glycation end products;

BW: body weight; WC: Waist Circumference; HC: Hip Circumference; TG: triglycerides; HDL-C: high-density lipoprotein cholesterol; DBP: Diatolic blood pressure; UA: Uric acid; HbA1c: Glycated hemoglobin;
